# No Significant Differences in Clinical and Radiographic Outcomes between PCL Retained or Sacrificed Kinematic Aligned Medial Pivot Total Knee Arthroplasty in Varus Knee

**DOI:** 10.3390/jcm11216569

**Published:** 2022-11-05

**Authors:** Fortunato Giustra, Francesco Bosco, Giorgio Cacciola, Salvatore Risitano, Marcello Capella, Alessandro Bistolfi, Alessandro Massè, Luigi Sabatini

**Affiliations:** 1 Department of Orthopedics and Traumatology, University of Turin, CTO Turin, Via Zuretti 29, 10126 Turin, Italy; 2 Orthopedics and Traumatology, Ospedale Cardinal Massaia Asti, Via Conte Verde 125, 14100 Asti, Italy

**Keywords:** total knee replacement, TKA, PCL, retain, sacrifice, medial pivot, MP, kinematic alignment, KA

## Abstract

In the last decades, several surgical techniques, such as medial pivot (MP) philosophy and kinematic alignment (KA), have been introduced in total knee arthroplasty (TKA) to improve patients’ outcomes. This retrospective study aims to evaluate the clinical, radiographic, and functional results of PCL preservation or sacrifice in KA MP-TKA. A consecutive series of 147 patients older than 60, with a minimum follow-up of two years, were treated with TKA for severe primary knee osteoarthritis (OA) at the Department of Orthopedics and Traumatology between 1 January 2019, and 1 July 2020. After excluding those not meeting the inclusion criteria, 64 patients were included in the study analysis. Regarding radiographic outcomes, no statistically significant difference was observed between patients with preserved or sacrificed PCL (*p* > 0.05). A slight improvement in Knee Society Score (KSS), knee and function score, and FJS was observed for the PCL-preserved group, although this superiority tendency was not statistically significant (*p* > 0.05). PCL-preserved MA MP-TKA reported a statistically significant result in only two questions on the FJS questionnaire (*p* < 0.05). A slight, non-statistically significant improvement in active ROM was found in the PCL-sacrificed group (*p* > 0.05). No interventions or revisions were reported in this case series for all treated patients at the final follow-up. No significant differences were described in clinical, radiographic, and functional outcomes in preserved or sacrificed PCL KA MP-TKA. Although not significant, a slight trend toward better clinical outcomes was reported in PCL-preserved KA MP-TKA.

## 1. Introduction

Total knee arthroplasty (TKA) is the international standard of care for end-stage knee osteoarthritis treatment. It is one of Europe and North America’s most widely performed elective orthopedic procedures [1,2]. Despite an optimal implant survival of more than 90% after 15 years, according to long-term studies and registry data [3,4], one in five patients is dissatisfied with surgical treatment [5,6]. There may be multiple reasons behind these results. Among them, sociodemographic, preoperative, intraoperative, and postoperative factors should be considered [5,6]. Several materials and surgical techniques have been introduced into clinical practice to improve patient satisfaction and long-term clinical and functional outcomes [7,8,9,10,11].

In the early 1990s, medial-pivot (MP) TKA was introduced to restore “natural knee kinematics.” The aim was to reproduce the physiologic ball-in-socket mechanism of the medial femoral condyle, while the lateral compartment, being less constrained, allowed an anteroposterior roll of the lateral femoral condyle during knee flexion-extension motion [12,13]. This implant design should avoid the “paradoxical” anterior rolling of the femoral condyles during knee flexion present in various prosthetic models such as the Posterior Stabilized (PS) or the Cruciate Retaining (CR), which reproduce non-physiological knee kinematics [14,15]. Several studies observed that MP-TKA reported comparable implant survival and better patient-reported outcome measures (PROMs) than other TKA designs [16,17,18].

In 2007, Stephen Howell theorized the kinematic alignment (KA) concept as a new TKA surgical approach to performing a resurface of the articular knee surfaces [19,20,21]. KA TKA is based on the three-axis theory of knee rotation, derived from biomechanical studies that proved that the epicondylar axis, on which mechanically aligned (MA) TKA is established, is different from the axis derived from the cylinders of best fit for the femoral condyles which underlie KA TKA. The three axes consist of the primary femoral axis on which the tibia flexes on a cylinder that best fits the articular surface; the secondary femoral axis, which represents the patella-femoral flexion-extension axis, is parallel to the first axis and about one centimeter more proximal. Finally, the longitudinal tibial axis, perpendicular to the previous one, describes the tibia’s internal–external rotation axis [5,19]. KA TKA aims to recreate the natural knee joint-line pre-arthritic alignment by restoring the three axes, resulting in natural knee kinematics [21]. Despite KA’s theoretical advantages, literature results are contradictory because some studies reported improved PROMs [22,23], while others described no significant differences between KA and MA TKA [24].

A proper flexion-extension gap and collateral ligament tension play a key role in improving implant survival and patient satisfaction in TKA [25]. Maintaining or sacrificing the posterior cruciate ligament (PCL) results in flexion-extension gap changes and varus/valgus laxity [26]. The PCL is the strongest intra-articular structure that prevents the posterior tibial translation in flexion and the internal rotation in flexion from becoming greater than 90° [27]. Some studies found no difference in varus/valgus laxity after PCL excision, but most were performed on cadavers [28,29]. In their prospective study, Kayani et al. reported that PCL excision significantly increased the flexion gap by an average of 2.4 mm (mm) medially and 3.3 mm laterally, which is higher than the extension gap of 1.3 mm medially and 1.2 mm laterally [30].

Since no comparative studies in the literature report the differences between KA MP-TKA preserving or sacrificing the PCL, it was hypothesized that patients who underwent KA MP-TKA and maintained the PCL could achieve better clinical and radiographic outcomes and the same function as patients where PCL was sacrificed.

## 2. Materials and Methods

A retrospective study was conducted on an initial consecutive series of 147 patients with severe knee osteoarthritis in whom a primary TKA was implanted at the Department of Orthopedics and Traumatology, C.T.O., Città della Salute e della Scienza, Turin, between 1 January 2019, and 1 July 2020.

### 2.1. Inclusion and Exclusion Criteria

The inclusion criteria were patients over 60 years of age with primary end-stage osteoarthritis (grades three and four of the Kellgren-Lawrence classification) [31] who underwent primary TKA according to KA-calibrated criteria [21] using a medial congruent (MC) TKA (Persona MC, by Zimmer Biomet) [7] with a minimum follow-up of two years. Patients under 60 years of age with secondary knee osteoarthritis that was not severe (grade one or two of the Kellgren-Lawrence classification) underwent primary TKA according to the mechanical alignment criteria, while those with non-medial congruent implants, valgus knee, follow-up of less than two years, previous osteotomies or ligament reconstructions, or previous severe traumatic surgical treatment around the knee were excluded from the present study.

### 2.2. Radiological Evaluation

As part of the preoperative planning, each patient was evaluated with an anteroposterior view, a lateral view, a Rosenberg view, a Merchant and Lauren view, and a full-length weight-bearing radiograph of the lower limb. Lower limb alignment was assessed preoperatively and postoperatively by calculating the hip-knee angle (HKA), mechanical distal lateral femoral angle (mLDFA), medial proximal tibia (MPTA), knee joint line orientation (KJLO), and tibial slope [32]. Two authors (FB and FG) completed the radiographic evaluation using the Modern Knee Society Radiographic Evaluation System established by Meneghini et al. [33].

### 2.3. Surgical Technique

All surgical procedures were performed by the same senior surgeon (LS), with Persona MC (from Zimmer Biomet), according to calibrated KA criteria [21]. The PCL was preserved in 35 patients, while it was sacrificed in 29 patients.

**Figure 1:**
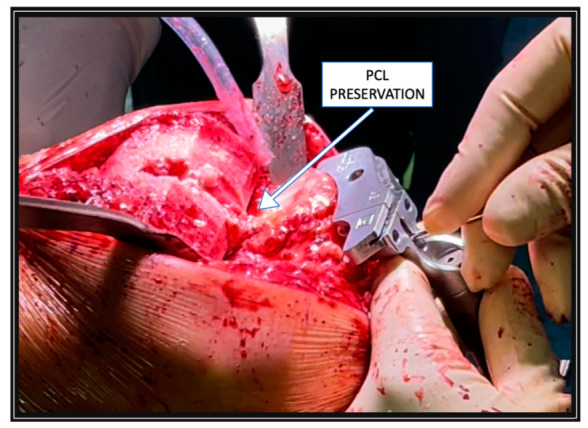
The posterior cruciate ligament (PCL) was preserved during tibial cutting.

Until August 2019, the PCL was always sacrificed in all treated patients. Starting in September 2019, considering the theoretical advantages of PCL preservation based on recent scientific evidence [34,35,36,37], such as increased anteroposterior stability and improved proprioception, we decided to retain the PCL in every case. In patients with tight flexion, space was evident after bone resections and gap balancing based on tibial recut was performed (Figure 1).

A median skin incision was performed, approximately three centimeters (cm) proximal to the femoral trochlea and four to five cm above the proximal tibia joint line, followed by a medial parapatellar arthrotomy to achieve adequate exposure. Bone cuts were made after adequate exposure and protection of the posteromedial corner, collateral ligaments, iliotibial bands, and popliteal tendon. According to KA criteria, a “femur first” bone resection was performed, followed by a tibial resection. The tibial cut was performed with a posterior tibial slope (PTS) of 5° as indicated by the manufacturer for the use of an MC liner. A calibrated KA with manual instrumentation was adopted in all cases [21].

After performing bone resections, the integrity of the PCL should be checked. At 90° of flexion, insert the narrowest spacer block; the medial compartment should be more constrained, while the lateral compartment should be looser, allowing 15° internal and external rotation.

Therefore, a slight varus/valgus movement (1 mm) should be observed with the knee fully extended. Hyperextension of 4°–6° is preferable to neutral extension, as in the natural knee. Finally, varus/valgus stability should be checked at 30° of flexion; the medial side should open no more than 1 mm and the lateral side no more than 3 mm. In case of extension, flexion, or mid-flexion imbalance, several strategies may be used to balance the knee without violating the KA principle [21,38,39].

### 2.4. Postoperative Rehabilitation Protocol

**Figure 2:**
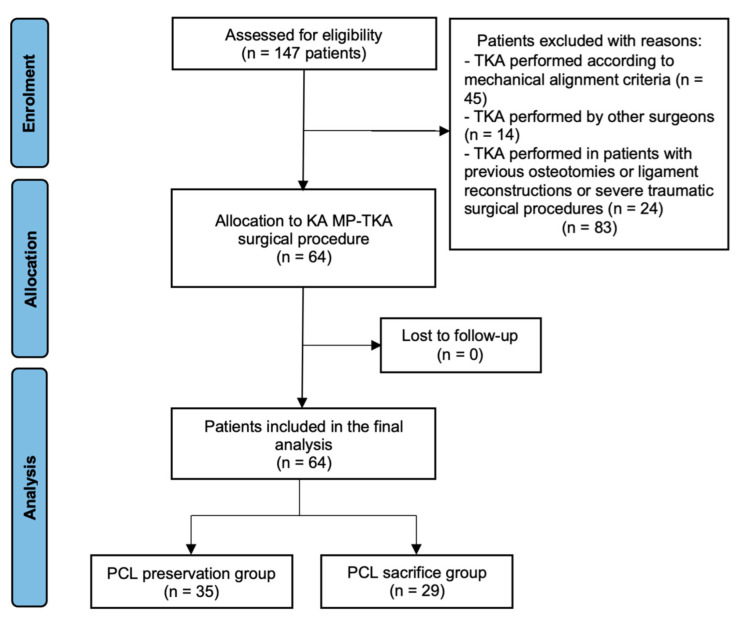
Flow diagram of study: all patients. TKA: total knee arthroplasty; KA MP-TKA: kinematic alignment medial pivot- total knee arthroplasty; PCL: posterior cruciate ligament.

The day after surgery, patients started to perform isometric exercises with crutches for muscle strengthening and full weight-bearing under the supervision of a physical therapist, following the hospital’s guidelines. Generally, twenty days after surgery, the use of crutches gradually decreased until the patient could walk without assistance. Afterward, patients were examined clinically and radiographically after one month, three months, six months, one year, and annually after the first year (Figure 2). Postoperative radiographs were requested to reveal any signs of implant loosening. All evaluations and measurements were performed with DICOM viewer and TraumaCad^®^ planning software (Brainlab, Munich, Germany).

### 2.5. PROMs

PROMs were measured using the Knee Society Score (KSS) knee score and function score with a range of 0 to 100, from worst to best [40], the Forgotten Joint Score (FJS) [41], the VAS, which measures pain at rest and mobility [42], and active range of motion (ROM). Scores were assessed before surgery and after one month, three months, six months, one year, and two years after surgery.

### 2.6. Data Extraction

Two authors (LS and GC) performed a data collection tool helped by two other authors (FB and FG). The following data were analyzed: preoperative and postoperative demographic, clinical and radiographic data, and complications of patients undergoing KA MP-TKA with or without PCL preservation.

### 2.7. Statistical Analysis

A paired *t*-test for continuous variables and a chi-square test for categorical variables was used. A univariate analysis was performed to assess the differences between patients before and after TKA. Using Student’s *t*-test for continuous variables and the chi-square test for categorical variables, differences in parameters between TKA cruciate-retaining and substituting KA TKA were investigated. A two-sized Mann-Whitney U test, at the significance level (alpha) 0.05, adjusted for multiplicity with Bonferroni correction, was used to detect sample size calculation. The prosthesis survival rate was analyzed using Kaplan-Meier analysis with a 95% confidence interval. Survival rates between cruciate-retaining and substituting KA TKA were compared using the log-rank test. TKA reoperations or revision for any reason were considered as the primary endpoint.

## 3. Results

### 3.1. Preoperative Demographic and Clinical Data

**Figure 3:**
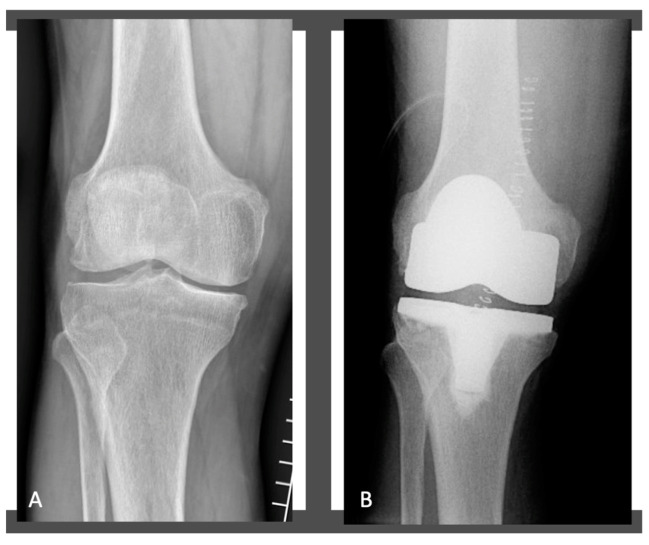
Radiographs of the patient’s knee before (**A**) and after KA MP-TKA (**B**).

After applying inclusion and exclusion criteria, we included 64 KA MP-TKAs for the final analysis (Figure 3). There were 37 women (57.8%) and 27 men (42.2%) with an average age of 73.4 ± 8.2 years. The average follow-up was 28.9 (24–33.5) months. PCL was retained in 35 patients (54.7%), while it was sacrificed in 29 cases (45.3%). There was no statistically significant difference (*p* > 0.05) in the demographic and preoperative clinical data and radiographic data between patients who retained or sacrificed the PCL (Table 1).

### 3.2. Preoperative and Postoperative Radiographic Measurements

The average preoperative HKA was 174.7° ± 4.4°, meaning that most knees were in varus. The average preoperative MPTA value was 86.6° ± 3.6°. The average preoperative LDFA was 88.4° ± 3.3°. The average preoperative tibial slope was 7.7° ± 4.9°. There was no significant difference (*p* > 0.05) between the preoperative value for patients who retained or sacrificed the PCL. The average postoperative HKA was 176.2° ± 3.9°; the average postoperative LDFA was 86.3° ± 3.4°. The average postoperative MPTA was 87.3° ± 2.2°; the average postoperative tibial slope was 5.4° ± 4.1°. We reported no significant difference (*p* > 0.05) in preoperative and postoperative radiographic outcomes between patients who retained or sacrificed the PCL (Table 2).

### 3.3. Preoperative and Postoperative Clinical Outcomes

After analyzing the outcome of the 64 KA MP-TKAs, we reported that the KSS knee score improved from an average of 48.6 ± 6.2 to an average of 94.5 ± 7.7 (*p* < 0.05). The average preoperative KSS function score improved from an average of 45.2 ± 5.8 to an average of 91.4 ± 7.4 (*p* < 0.05). Sample size calculation analysis showed that the sample size of the groups reached 80% power in the test of the KSS knee and function scores, which were 16 and 20, respectively. The average active ROM improved from an average of 114.5° ± 11.3° to an average of 118.7° ± 11.7° (*p* < 0.05). The average postoperative FJS was 63.3 ± 19.8 (*p* < 0.05). All the scores reported a statistically significant difference between preoperative and postoperative values (*p* < 0.05). We reported a slight improvement in KSS knee score, function score, and FJS for the PCL preservation group, but this trend of superiority, compared with the PCL sacrifice group, was not significant (*p* > 0.05). We reported a slight improvement in active ROM in patients in the PCL sacrifice group, but the difference with the PCL preservation group was not significant (*p* > 0.05) (Table 3).

The average FJS was higher in the PCL preservation group, but the difference with the PCL sacrifice group was not statistically significant (*p* > 0.05). A statistically significant result was reported for two questions of the FJS questionnaire for the PCL preservation group (*p* < 0.05). The first question was, “Are you aware of your artificial joint when walking on an even ground?” and the second question was, “Are you aware of your artificial joint standing from a low sitting position?” (Table 4).

### 3.4. Complications and Survivorship

A postoperative complication was reported in five KA MP-TKAs (7.8%). Three patients in the PCL preservation group (8.6%) reported reduced ROM. Two patients had an extension gap of about 10°, and one had a maximum flexion of 95°. Two patients in the PCL sacrifice group reported a reduced postoperative ROM. In one case, the patient had a gap of 10° in extension, and in the second case, the patient had a maximum flexion of 90°. Despite the limited ROM, no patients required further surgical procedures and were satisfied. Implant survival at a mean follow-up of 28.9 (24–33.5) months was 100% (95%CI, 100–100) in both groups with preserved and sacrificed PCL. No reoperations or revisions were reported in the present case series at the final follow-up. Table 5 shows the clinical and radiographic scores of patients who reported complications.

## 4. Discussion

The most important finding of this paper is that PCL preserving or sacrificing does not lead to significant differences in radiographic, clinical outcomes and complication rates among patients undergoing KA MP-TKA. Slightly better KSS function scores and overall FJS were observed in the PCL-preserved group and not in the PCL-sacrificed one, but these differences were not statistically significant. A higher ROM, although not significant, was observed in the PCL-sacrificed group. The only statistically significant difference, with better results in patients undergoing PCL-preserved KA MP-TKA, was observed in the mean score of two FJS questions, although the total FJS score reported no significant difference between the two groups.

Several studies have compared patients’ outcomes with PCL-preserved or sacrificed TKA; generally, no significant differences were described between the two groups of patients [34,43,44,45]. Thippanne et al. [43] reported no statistically significant differences for FJS between 169 PCL-retained TKAs and 178 PCL-sacrificed TKAs. Similarly, Bieganowski et al. [44] compared mean postoperative FJS in 671 TKAs; 236 were treated with a PCL preservation implant and 435 were treated with a posterior stabilized prosthesis. The authors also evaluated the influence of linear constraint level on knee proprioception. Significant differences in FJS were observed according to linear constraint; specifically, higher levels of constraint were associated with lower FJS in posteriorly stabilized TKAs. Nevertheless, with the same constraint level, no significant differences were established between PCL-retained and posteriorly stabilized TKAs. In their comparative study, Stronach et al. [45] reported no differences in postoperative ROM between MA PCL-retained and PCL-sacrificed TKAs.

Starting in September 2019, the PCL was preserved in all patients undergoing TKA by correcting any tight extension or flexion gaps after the femoral and tibial cut on the bases of the calipered KA [39]. To verify tibial cut correctness, place the knee at 90° of flexion and insert the tightest-fitting spacer block. Usually, the spacer should provide internal–external rotation with a pivot in the center of the medial compartment, demonstrating the trapezoidal flexion space recovery. The trial component could verify about 15° of tibia internal/external rotation. If the tibial cut was performed with a lower PTS than that of a pre-arthritic knee, the flexion space is too tight and a tibial recut needs to be performed to increase the PTS and restore the correct flexion gap. To check the extension gap appropriateness, place the spacer block in full extension. A minimal varus/valgus laxity should be observed, and in the case of lateral or medial openings greater than 2 mm, a tibial recut is mandatory [35,36]. Then, position the trial component and repeat the varus/valgus stress evaluation in full extension and at 30° of knee flexion. The medial compartment should open no more than 1 mm and the lateral compartment should have a maximum opening of 2–3 mm. For a knee that is stable in full extension but loose in flexion, consider reducing the posterior slope by performing an anterior tibial cut of 2–3 mm.

Howell et al. [37] demonstrated that a PCL retaining technique should be performed even in valgus deformity, in which the PCL is tighter than in the neutral or varus-aligned knee. Other authors, such as Stornach et al. [45], decided to maintain or sacrifice the PCL intraoperatively based on the flexion and extension gap tightness at the time of implant trailing. The PCL was released when an anterior tibia translation at 90° of knee flexion was detected because this condition was suggestive of a tight flexion gap [45].

Several limitations should be considered in this study. First, it is a retrospective evaluation with limitations related to the study design. Second, the number of PCL-sacrificed TKAs was lower than the PCL-preserved TKAs. Third, the TKA follow-ups were relatively short. Fourth, the same prosthetic implant was used in all patients. Finally, the authors attempted to conduct the paper as closely as possible to a consecutive series, mainly excluding patients undergoing MA TKA, previous surgeries around the knee, or who underwent TKA not performed by senior surgeons; however, this could lead to a possible sampling bias. Further studies with larger samples, longer follow-ups, and different prosthetic implants may confirm these results.

## 5. Conclusions

The KA MP-TKA provides optimal clinical and radiographic results and an acceptable postoperative complication rate with both preserved and sacrificed PCL. Only slightly better PROMs in PCL-preserved KA MP-TKAs were reported in this study, but the difference was not statistically significant. Considering both groups’ average FJS and PROMs, PCL preservation or sacrifice should be related to the TKA flexion gap and surgeon preference.

## Figures and Tables

**Table 1:** Preoperative demographic and clinical data of patients undergoing KA MP-TKA with or without PCL preservation.

Parameters	Total Knees			PCL Preservation Group			PCL Sacrifice Group			*p* Value
	N° (%)	Mean	SD	N° (%)	Mean	SD	N° (%)	Mean	SD	
Total patients	64 (100%)			35 (54.7%)			29 (45.3%)			
Women	37 (57.8%)			21 (56.8%)			16 (43.2%)			0.697
Age (years)		73.4	8.2		72.8	9.1		74.1	7.9	0.787
BMI (Kg/m^2^)		26.4	4.4		25.9	3.9		26.7	4.6	0.656
KSS knee Score		40.3	11,2		41.1	10.8		39.9	11.4	0.766
KSS function Score		38.5	9.9		39.9	10.4		37.5	9.2	0.645

N°: number of patients; %: percentage; PCL: posterior cruciate ligament; SD: standard deviation; BMI: body mass index; Kg/m^2^: kilogram per square meter; KSS: Knee Society Score.

**Table 2:** Preoperative and postoperative radiographic data in the PCL preservation and PCL sacrifice group.

Measurements	Preoperative							Postoperative						
	Total Knees		PCL Preservation Group		PCL Sacrifice Group		*p* Value	Total Knees		PCL Preservation Group		PCL Sacrifice Group		*p* Value
	Mean	SD	Mean	SD	Mean	SD		Mean	SD	Mean	SD	Mean	SD	
HKA	174.4°	4.4°	173.9°	3.9°	174.7°	4.5°	0.565	176.2°	3.9°	175.9°	3.8°	177°	3.4°	0.772
MPTA	86.6°	3.6°	86.8°	3.7°	85.8°	3.9°	0.772	86.3°	3.4°	85.8°	3.2°	86.6°	3.6°	0.564
LDFA	88.4°	3.3°	88.2°	3.6°	88.7°	3.5°	0.688	87.3°	2.2°	87.1°	2.5°	87.5°	2.1°	0.423
PTS	7.7°	4.9°	7.3°	4.7°	7.9°	5.1°	0.881	5.4°	4.1°	5.2°	3.8°	5.7°	4.2°	0.776

PCL: posterior cruciate ligament; SD: standard deviation; °: degree; HKA: hip-knee-ankle angle; MPTA: medial proximal tibial angle; LDFA: lateral distal femoral angle; PTS: posterior tibial slope.

**Table 3:** Preoperative and postoperative clinical data in the PCL preservation and PCL sacrifice group.

PROMs	Preoperative							Postoperative						
	Total Knees		PCL Preservation Group		PCL Sacrifice Group		*p* Value	Total Knees		PCL Preservation Group		PCL Sacrifice Group		*p* Value
	Mean	SD	Mean	SD	Mean	SD		Mean	SD	Mean	SD	Mean	SD	
KSS knee score	48.4	6.2	47.2	5.8	48.7	6.4	0.778	94.5	7.7	96.7	8.1	93.1	5.9	0.343
KSS function score	45.2	5.8	46.1	6.1	44.8	5.7	0.664	91.4	7.4	93.2	7.9	89.8	7.7	0.289
FJS	N/A	N/A	N/A	N/A	N/A	N/A	N/A	60.3	18.4	63.3	19.8	59.7	18.1	0.051
Active ROM	114.5°	11.3°	113.8°	10.8°	115.2°	11.5°	0.667	118.7°	11.7°	116.4	11.8°	125.4°	12.1°	0.074

PROMs: patient-reported outcome measures; PCL: posterior cruciate ligament; SD: standard deviation; °: degree; KSS: Knee Society Score; FJS: Forgotten Joint Score; ROM: range of motion; N/A not available.

**Table 4:** Forgotten Joint Score applied to patients included in the present study.

Q: Are You Aware of You Artificial Joint When?	PCL Preservation Group	PCL Sacrifice Group	*p* Value
	Mean ± SD	Mean ± SD	
1. In a bed at night	2.48	2.55	0.331
2. Sitting in a chair > 1 h	2.89	2.61	0.087
3. Walking for > 15 min	2.65	2.78	0.303
4. Taking a bath/shower	1.78	1.9	0.432
5. Travelling in a car	2.44	2.71	0.087
6. Climbing stairs	2.78	3.1	0.323
7. Walking on uneven ground	2.5	3.1	*0.045*
8. Standing from low sitting position	2.48	3.12	*0.041*
9. Standing for a long period of time	2.88	2.92	0.512
10. Doing housework/gardening	3.01	2.99	0.445
11. Taking a walk/hike	2.77	3.02	0.061
12. Doing your favorite sport	2.98	3.11	0.322
Total Score	59.5 ± 21.1	55.7 ± 19.3	0.064

Q: Question; PCL: posterior cruciate ligament; SD: standard deviation; h: hour; min: minutes. Significant *p*-values are shown in bold italics.

**Table 5:** Complications after KA MP-TKA.

Patients with Complications	Age (Years)	Sex	PCL Preservation (Y/N)	Postoperative KSS Knee Score	Postoperative KSS Function Score	Postoperative FJS	Active ROM	Complications	Need to TKA Revision or Reoperation (Y/N)
1	73	F	Y	88.2	86.4	58.1	93°	Extension deficit	N
2	68	M	Y	89.5	87.1	55.4	94°	Flexion deficit	N
3	76	M	N	92.1	89.8	59.4	96°	Extension deficit	N
4	74	F	N	85.2	81.4	60.1	87°	Extension deficit	N
5	77	F	y	79.1	76.4	55.1	91°	Flexion deficit	N

PCL: posterior cruciate ligament; Y: Yes; N: Not; KSS, knee society score; FJS: Forgotten Joint Score; ROM: range of motion; TKA: total knee arthroplasty.

## Data Availability

The data presented in this study are available in the article.
